# Evasion of Neutrophil Killing by *Staphylococcus aureus*

**DOI:** 10.3390/pathogens5010032

**Published:** 2016-03-17

**Authors:** Will A. McGuinness, Scott D. Kobayashi, Frank R. DeLeo

**Affiliations:** Laboratory of Bacteriology, Rocky Mountain Laboratories, National Institute of Allergy and Infectious Diseases, National Institutes of Health, 903 South 4th Street, Hamilton, MT 59840, USA; will.mcguinness@nih.gov (W.A.M.); kobayashis@niaid.nih.gov (S.D.K.)

**Keywords:** innate immunity, neutrophil, phagocytosis, PMN, *Staphylococcus aureus*

## Abstract

*Staphylococcus aureus* causes many types of infections, ranging from self-resolving skin infections to severe or fatal pneumonia. Human innate immune cells, called polymorphonuclear leukocytes (PMNs or neutrophils), are essential for defense against *S. aureus* infections. Neutrophils are the most prominent cell type of the innate immune system and are capable of producing non-specific antimicrobial molecules that are effective at eliminating bacteria. Although significant progress has been made over the past few decades, our knowledge of *S. aureus*-host innate immune system interactions is incomplete. Most notably, *S. aureus* has the capacity to produce numerous molecules that are directed to protect the bacterium from neutrophils. Here we review in brief the role played by neutrophils in defense against *S. aureus* infection, and correspondingly, highlight selected *S. aureus* molecules that target key neutrophil functions.

## 1. Introduction

*Staphylococcus aureus* is adept at circumventing destruction by the human innate immune system, and the bacterium imposes a significant disease burden on at-risk and otherwise healthy populations worldwide [1]. *S. aureus* commonly resides as a quiescent commensal within the human nares, and, as such, is a frequent cause of opportunistic infections [2]. Indeed, *S. aureus* has remained a leading cause of healthcare and community associated infections in the United States over the past 60 years. The organism causes a broad array of diseases and syndromes, such as endocarditis, toxic-shock syndrome, bacteremia, soft-tissue infections, surgical-site infections, and pneumonia [3,4,5,6]. In addition, the pathogen is notorious for its ability to acquire antibiotic resistance, and *S. aureus* increasingly undermines treatment regimens already hampered by a substantial lack of novel therapeutics [7,8]. Taken together, the propensity for *S. aureus* to develop antibiotic resistance and cause significant morbidity, underscores the importance of developing new therapeutic approaches for treatment of infections.

Polymorphonuclear leukocytes (PMNs or neutrophils) are the most prominent cellular component of the host innate immune response to bacterial and fungal infections. The vast majority of healthy individuals are seemingly protected against severe *S. aureus* infections, and this characteristic is attributed largely to the role played by neutrophils in host defense. Indeed, individuals with neutrophil defects are highly susceptible to severe and life-threatening *S. aureus* infections [9]. This review highlights the role of neutrophils in innate immunity, and specific mechanisms used by *S. aureus* to circumvent elimination by PMNs.

## 2. Neutrophils in Host Defense

Neutrophils are the primary cellular constituent of the human innate immune system and are essential for host defense against invading microbial pathogens. PMNs originate and mature in the bone marrow, and are subsequently released into the peripheral vasculature. High numbers of neutrophils are maintained in circulation to facilitate rapid recruitment to infected tissue. Intravascular circulating neutrophils comprise approximately half of the entire granulocyte population, whereas marginated neutrophils maturing in the bone marrow or resident in organ systems make up the remaining half [10,11]. Mature neutrophils enter the vasculature from the bone marrow at a rate of approximately 10^9^ cells/kg body weight every day in a healthy human adult [10,11]. Circulating neutrophils, by sheer force of presence, are poised for recruitment by chemotactic signals to areas of infection. The large pool of circulating PMNs is available to support the inflammatory response to infection.

Circulating neutrophils are recruited to infection sites through chemotactic signals produced by host cells and shed and/or secreted bacterial molecules. The ability of neutrophils to rapidly respond to invading microorganisms is a key facet of innate immunity. Neutrophils are highly sensitive to chemotactic signals that signify infection and promote migration. Host-derived chemotactic factors, such as interleukin 8, GRO-alpha, granulocyte chemotactic protein 2, and complement component C5a are potent proinflammatory mediators that are used to recruit additional PMNs to areas of infection [12,13,14]. Further, PMN migration can be elicited directly or indirectly by bacteria-derived stimuli, such as lipoteichoic acid or *n*-formyl peptides [15,16,17,18,19,20]. At sites of infection, a diversity of resident cells contribute to production of chemotactic factors including macrophages, endothelial, and epithelial cells [21,22,23].

Neutrophil recruitment from the bloodstream to infected tissue is governed by a series of complex signal transduction cascades that ultimately control the dynamics of PMN interaction with vascular cells and regulate chemotaxis [12,13]. The neutrophil recruitment process can be broken into four steps: Rolling adhesion, integrin activation, firm adhesion, and transmigration. PMN recruitment is initiated by inflammatory mediators, and is followed by the expression of surface ligands on the neutrophil plasma membrane [12,13,24]. During rolling adhesion, PMNs travel through the bloodstream by shear force within blood vessels, adherence to the blood vessel walls is maintained by repetitive ligand-receptor binding mediated by selectins allowing for reversible and bidirectional modulation of the binding process of neutrophils to endothelial cells [12,13,24]. Neutrophil-endothelial cell (blood vessel) adhesion interactions–mediated by expression of lymphocyte function-associated antigen-1 (LFA-1)–shift to a higher affinity state following stimulation by proinflammatory mediators. LFA-1 binds to intracellular adhesion molecule (ICAM)-1 and ICAM-2 present on endothelial cells, resulting in neutrophil arrest [13,24]. LFA-1 integrin clustering on the neutrophil plasma membrane increase the avidity of the binding process and stimulates cytoskeletal/receptor rearrangements that induce PMN morphological changes [24]. As the PMN morphology shifts from spherical to flat, shear force is no longer capable of initiating neutrophil movement through the blood vessel [12,13]. Following firm attachment to the endothelium, neutrophil transmigration from the endothelium into infected tissue is facilitated by several leukocyte and endothelial cell molecules including CD31, CD11b/CD18, CD47, and CD44 [12,13].

**Figure 1:**
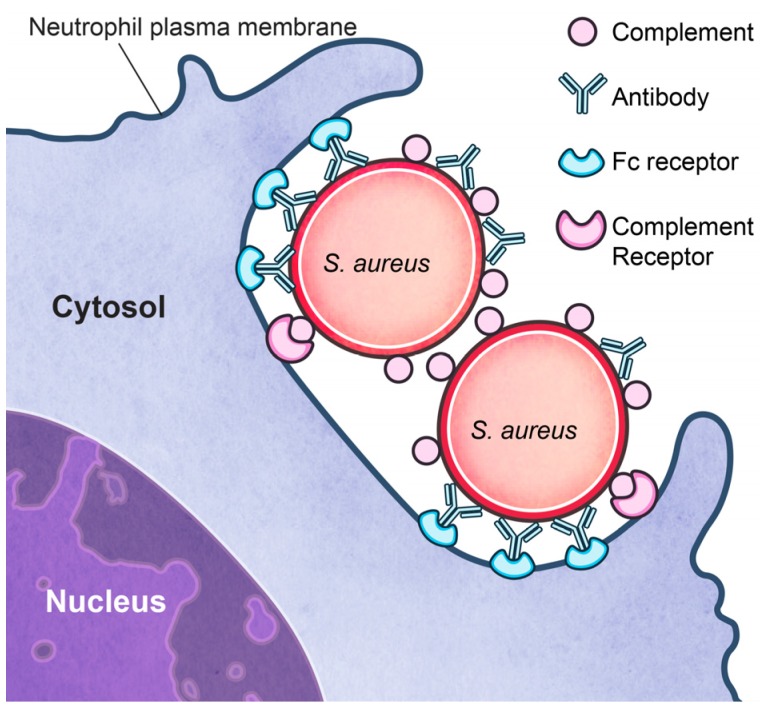
Neutrophil phagocytosis of *S. aureus*. *S. aureus* opsonized with serum complement components (complement) and specific antibody (antibody) are efficiently bound by complement receptors and Fc receptors on the surface of neutrophils. Ligation of these receptors drives the process of phagocytosis.

Neutrophils play a primary role in host defense and remove pathogens through a process known as phagocytosis. Phagocytosis is initiated by recognition and binding of bacteria, and is followed by internalization. Recognition of microbial pathogens is determined by pattern recognition receptors (PRRs) and opsonin receptors. PRRs bind microbe-associated molecules known as pathogen-associated molecular patterns (PAMPs), and PRRs are an essential component of the host-immune surveillance. The composition of PRRs includes a broad array of receptors, such as membrane-bound toll-like receptors (TLRs), that identify highly conserved bacterial surface and secreted molecules. PRR ligation induces intracellular signal transduction cascades that trigger enhanced phagocytosis and cytokine production, albeit pathogen recognition by PRRs alone is generally insufficient to stimulate phagocytosis [25,26,27]. The efficiency of phagocytosis is enhanced when invading microorganisms are opsonized with host serum molecules such as complement and antibody. Specific IgG, when bound to microbial surfaces, directs the deposition of serum complement. Distinct IgG and complement receptors on the neutrophil directly mediate uptake of opsonized microbes [28,29] (Figure 1). Fc receptor binding leads to the induction of highly complex signal transduction cascades that direct membrane reorganization, actin polymerization, and cytoskeletal rearrangements that are essential for phagocytosis. Electrophysiological alterations in transmembrane potential elicit lipid signaling cascades and actin polymerization that contribute to the advancing membrane cup. Complete microbial engulfment is accomplished when the membrane cup entirely surrounds the pathogen and closes to form a nascent phagosome.

**Figure 2:**
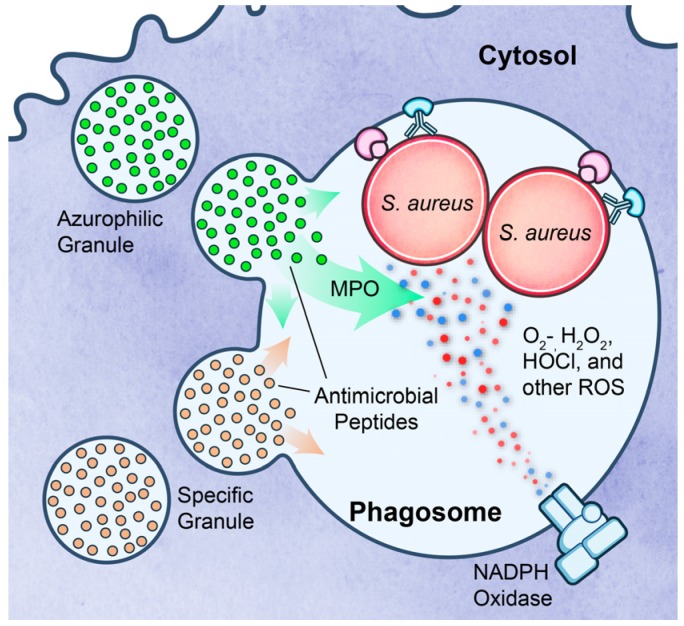
Neutrophil microbicidal mechanisms. Bacteria are exposed to PMN-derived oxygen-dependent and –independent factors within the phagosome. See text for details.

Neutrophils use both oxygen-dependent and oxygen-independent processes to kill invading microbial pathogens (Figure 2). Phagocytosis of bacteria triggers assembly of a membrane-bound NADPH-dependent oxidase on PMN phagosomes [30,31,32]. The NADPH oxidase complex generates high levels of reactive oxygen species (ROS) in activated neutrophils [32,33]. The NADPH oxidase functions by shuttling electrons across the phagosomal membrane from cytosolic NADPH to intraphagosomal molecular oxygen to produce O^2−^. The superoxide anion is readily converted to hydrogen peroxide by superoxide dismutase. Notably, hydrogen peroxide and other secondary oxygen derivatives such hydroxyl radical, chloramines and hypochlorous acid (HOCl), which is generated by the myeloperoxidase-halide system, contribute to PMN microbicidal activity [33,34]. Neutrophil oxygen-independent microbicidal activity is effected by numerous degradative enzymes and cationic peptides contained in granules (primary, secondary, and tertiary) and secretory vesicles. Following phagocytosis, granules are trafficked to and fuse with PMN phagosomes in a process called degranulation [35,36,37]. Although the composition of granules varies by type, collectively they enrich the vacuole lumen with microbicidal agents such as cathepsins, elastase, proteinase-3, defensins, lysozyme, azurocidin and bacterial permeability increasing protein [35,36,38]. Collectively, PMN oxygen-dependent and oxygen-independent microbicidal systems are highly efficient for elimination of invading bacterial pathogens.

The rapid influx of large numbers of neutrophils to sites of infection is a hallmark of the acute inflammatory response. The combination of ROS, degradative enzymes, and cationic peptides create a potent microbicidal environment confined within neutrophil phagosomal membranes. Inasmuch as these molecules do not discriminate between host and microbe targets, the potential of host-tissue damage is high should the neutrophil undergo inappropriate cell lysis [39]. To mitigate tissue damage and control inflammation, activated neutrophils rapidly undergo apoptosis and effete cells are recognized and safely removed by macrophages through a process known as efferocytosis. Resolution of acute inflammation is coordinated through the activities of proresolving lipid mediators, such as resolvins, protectins, and maresins [40].

## 3. Evasion of *S. aureus* Killing by Neutrophils

**Figure 3:**
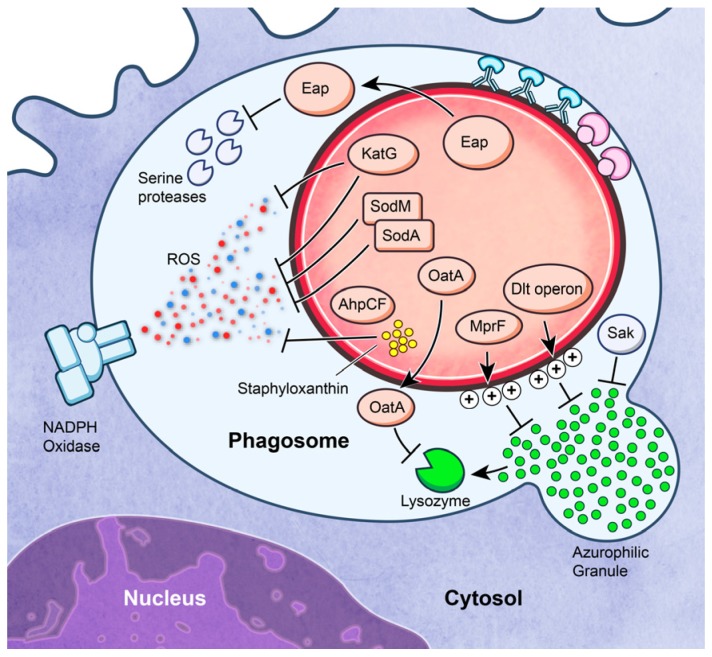
*S. aureus* evasion of killing by neutrophils. *S. aureus* produces several secreted and surface-bound molecules that are capable of interfering with neutrophil function. See text for details.

*S. aureus* is capable of producing numerous freely secreted and surface-bound molecules that have potential to alter and/or limit the functional capacity of neutrophils. *In vitro*, these molecules have been shown to inhibit neutrophil recruitment, bacterial binding and phagocytosis, and killing by ROS and antimicrobial peptides (AMPs) (reviewed in [41,42]) (Figure 3). Here, we review some of the *S. aureus* molecules and processes that contribute to evasion of killing by neutrophils.

### 3.1. Inhibition of Neutrophil Recruitment

Chemotaxis inhibiting protein of *S. aureus* (CHIPS) is a freely secreted protein that inhibits neutrophil and monocyte chemotaxis [43,44]. CHIPS binds directly to the C5a and formyl peptide receptors (FPRs), and thereby inhibits phagocyte recruitment [44]. The gene encoding CHIPS, along with those for 3 other immune evasion molecules—staphylococcal complement inhibitor (SCIN), staphylokinase (SAK), and *S. aureus* enterotoxin A or P (SEA or SEP)—is located on a mobile genetic element known as a β-hemolysin-converting bacteriophage [45]. SAK binds and inhibits the ability of neutrophil defensins to kill bacteria [46], and SEA or SEP are superantigens with known capacity to modulate phagocyte function [47,48,49]. Collectively, the genes encoding these molecules comprise an immune evasion cluster (IEC) that is present in many *S. aureus* strains tested to date [45]. More recently, Stapels *et al.* reported that *S. aureus* extracellular adherence protein (Eap) and two homologs, EapH1 and EapH2, inhibit neutrophil elastase, proteinase-3, and cathepsin G [50]. These neutrophil serine proteases can cleave *S. aureus* immune evasion molecules, such as SCIN and CHIPS, and this process is blocked by the Eap molecules [51]. The extent to which these molecules contribute to pathogenesis in humans remains to be determined.

There is also a family of staphylococcal-like proteins (Ssls) that has been reported to function as immune evasion molecules. For example, Ssl7 binds host IgA and complement component C5, inhibiting generation of C5a, phagocytosis, and production of phagocyte reactive oxygen species [52,53]. In addition, Bestebroer *et al.* found that Ssl7 blocked neutrophil recruitment in a mouse model of inflammation [52]. This same research group reported Ssl5 associates with P-selectin glycoprotein ligand-1 (PSGL-1, also known as CD162) and inhibits neutrophil rolling *in vitro* [54]. In subsequent studies, they demonstrated Ssl5 bound the amino terminus G protein-coupled receptors, thereby inhibiting leukocyte activation by host inflammatory molecules such as cytokines [55].

*S. aureus* produces at least five molecules that inhibit serum complement convertases—host enzyme complexes important for a fully functional complement system. The activity of host C3 convertase is essential for the opsonization of microbes with complement components such as C3b and iC3b, which promote phagocytosis by complement receptors. Moreover, activation of the complement system ultimately generates C5a, a neutrophil chemoattractant. Staphylococcal complement inhibitor (SCIN) and homologues (SCIN–B and SCIN–C), extracellular fibrinogen-binding protein (Efb), and extracellular complement-binding protein (Ecb), bind and inhibit the C3 convertase (SCIN proteins), or alternatively, C3b–containing convertases and C5 convertases (Efb and Ecb) [56,57]. The interaction of these *S. aureus* molecules with the host complement system inhibits leukocyte recruitment and neutrophil phagocytosis of *S. aureus in vitro* and *ex vivo* in human blood [58,59]. Although SCIN and homologues are human-specific, and thus cannot be tested effectively in animal infection models, Efb has been shown to inhibit phagocytosis of *S. aureus* in a mouse infection model [59]. The ability of Efb to block phagocytosis is independent of its ability to inhibit complement. Rather, Efb forms a complex with fibrinogen and C3b on the microbe surface, and in doing so blocks phagocytosis [59]. Jongerius *et al.* found that SCIN-B, SCIN-C, Efb and Ecb are located within a segment of the *S. aureus* genome that also encodes alpha-hemolysin (Hla), Ssl12, Ssl13, Ssl14, FPR-like 1 inhibitory protein (FLIPr), and a FLIPr homologue (FLIPr-like) [57]. Inasmuch as these genes contribute to *S. aureus* evasion of host defense, this segment of the genome was named immune evasion cluster 2 (IEC-2) [57].

Laarman *et al.* recently demonstrated that staphopain A (ScpA), a secreted *S. aureus* cysteine protease, inhibits *in vitro* neutrophil chemotaxis mediated by CXCR2 [60]. ScpA cleaves an N-terminal region of CXCR2, and this phenomenon blocks signal transduction triggered by key proinflammatory chemokines such as IL-8 and GROα [60]. The redundancy of molecules directed to inhibit neutrophil chemotaxis and complement activation is curious, especially for a commensal microbe that infrequently causes severe infections in otherwise healthy individuals.

### 3.2. Inhibition of Phagocytosis?

In addition to the complement inhibitors discussed above, several other *S. aureus* molecules have been shown to inhibit neutrophil phagocytosis *in vitro* or in whole blood. For instance, *S. aureus* protein A (SpA) and the second binding protein of immunoglobulin (Sbi) associate with the Fc-region of immunoglobulin and can inhibit phagocytosis *in vitro* [61,62,63,64]. *S. aureus* capsule polysaccharide [65,66], clumping factor A [67], and iron-regulated surface determinant protein H (IsdH) [68], have also been shown to inhibit *S. aureus* phagocytosis *in vitro*. *S. aureus* strains lacking at least two of these molecules—e.g., those lacking Spa and ClfA—are ingested to a greater extent by neutrophils than those lacking only one of these molecules [67]. It is noteworthy that none of these molecules, either singly or collectively, inhibit *in vitro* uptake by neutrophils completely. The ability of these molecules to block uptake is also probably dependent on assay conditions. The vast majority of the studies of *S. aureus* phagocytosis by neutrophils have been performed with cells in suspension. The extent to which phagocytosis assays with neutrophils in suspension approximate the interaction of bacteria with neutrophils in the bloodstream is unclear. It has long been known that neutrophils are largely responsible for clearance of *S. aureus* from the bloodstream in animal infection models [69]. However, neutrophils in suspension are relatively inefficient at phagocytosis, and published studies have shown that blood-borne bacteria are largely cleared by phagocytes (neutrophils) in tissues such as the liver [70]. Importantly, phagocytosis of *S. aureus* by adherent human neutrophils—which are representative of phagocytes at sites of infection—is highly efficient and seemingly unimpeded by anything produced by the microbe [71,72]. The rapid ingestion of *S. aureus* by neutrophils was noted by Rogers and Tompsett many years ago, and they also found that phagocytosis of *S. aureus* was significantly greater than that of *Klebsiella pneumoniae*, group A streptococcus, or *Streptococcus pneumoniae* [69,73]. Therefore, the role of *S. aureus* “antiphagocytic” molecules in the pathogenesis of human infections remains unclear. The problem with *S. aureus* and human infections is not inefficient phagocytosis (David Rogers had an interesting view on the topic [74]). Rather, a key issue is that some of the ingested bacteria are not killed by the microbicidal systems that operate within the neutrophil phagosome [71,73,75,76]. These ingested microbes ultimately cause lysis of neutrophils and, in turn, escape and disseminate [71,75,77].

### 3.3. Survival after Phagocytosis

*S. aureus* has the capacity to produce molecules that can moderate the cytotoxic effects of ROS and AMPs. Survival of *S. aureus* inside the phagosome is directly linked to the ability to mitigate the effects of host–derived ROS and AMPs. Superoxide dismutases (SodA and SodM), catalase (KatA), alkyl hydroperoxide reductase (AhpCF), and staphyloxanthin (encoded by *crtOPQMN*), have been shown to protect *S. aureus* from ROS [78,79,80]. *S. aureus* also mitigates the effects of ROS by means of proteins and enzymes involved in metal ion homeostasis (reviewed by Cassat and Skaar [81]). Although these systems moderate the bactericidal effects of ROS to some extent, it is well known that the NADPH oxidase of phagocytes is essential for defense against *S. aureus* infections. Individuals with chronic granulomatous disease, a disorder caused by inherited deficiency of one or more protein components of NADPH oxidase, are highly susceptible to severe *S. aureus* infections in the absence of antibiotic prophylaxis [82]. Most evidence to date indicates secondarily-derived ROS, such as HOCl produced by the MPO-halide system (rather than superoxide or H2O2 directly), contribute to killing of ingested *S. aureus* [83,84,85].

The *dlt* operon of *S. aureus* (*dltABCD*) encodes a cell envelope modification system that attaches d-alanine to cell wall teichoic acids [86]. Incorporation of d-alanine into teichoic acids decreases the negative charge on the bacterial surface and limits susceptibility of the microbe to cationic AMPs, including human neutrophil alpha-defensins [86,87]. Collins *et al.* used a mouse infection model to demonstrate that the *dlt* operon is important for *S. aureus* virulence [87]. Multiple peptide resistance factor (MprF) of *S. aureus* transfers l–lysine to phosphatidylglycerol to produce lysylphosphatidylglycerol, which contributes to neutralization of the negative charge of the bacterial cell surface [88]. *S. aureus* strains with *mprF* genetically inactivated are significantly more susceptible to killing by human neutrophils, a phenomenon linked to increased binding to host AMPs [88]. *S. aureus* *mprF* mutants also have a decreased virulence phenotype in mouse infection models, which further underscores the importance of the resistance to AMPs in *S. aureus* pathogenesis [88]. In general, DltABCD and MprF–mediated resistance to cationic AMPs works by charge neutralization—*i.e.*, the AMPs no longer interact with the charge-neutral membrane of the microbe. There are several other notable molecules and immune evasion systems utilized by *S. aureus* to promote survival after phagocytosis by neutrophils. For example, lysozyme is a prominent antimicrobial agent present in neutrophil granules. It functions as an antibacterial agent by hydrolyzing beta-glyosidic linkages in cell wall peptidoglycan. Notably, *S. aureus* is completely resistant to lysozyme. This a characteristic conferred by *O*-acetylation of peptidoglycan, a process catalyzed by a protein known as OatA [89].

### 3.4. Lysis after Phagocytosis

*S. aureus* is capable of producing an extensive arsenal of cytolytic toxins. Several of these molecules, including alpha-type phenol soluble modulins (PSMα) [90], Panton-Valentine leukocidin (PVL) [91,92,93], leukocidin GH (LukGH; also named leukocidin AB (LukAB)) [94,95], and gamma-hemolysin (Hlg) [96], cause osmotic lysis of neutrophils. The role played by these molecules as secreted cytolysins in the pathogenesis of *S. aureus* infections is an interesting topic, but outside of the scope of this review. Here we focus our brief discussion on the contribution of molecules that contribute to lysis of neutrophils after phagocytosis.

Rogers and colleagues discovered that *S. aureus* within granulocytes can be trafficked to tissues and promote low-grade persistent infection [69,74]. This same phenomenon was reported many years later by Gresham and colleagues using a mouse infection model [75]. Rogers and Tompsett were among the first to show that *S. aureus* causes lysis of neutrophils after phagocytosis [73]. Voyich *et al.* reported that prominent community-associated MRSA strains such as USA300 and USA400 have high capacity to cause rapid lysis of human neutrophils after phagocytosis [71]. Recent work has shown that LukGH/AB and PSMα to contribute to lysis of human neutrophils after phagocytosis [94,97]. However, the contribution of these molecules is strain-dependent or partial, and therefore cannot explain the phenomenon in full. Studies by Kobayashi *et al.* indicated that *S. aureus*—in this case USA300—is contained within the phagosome to the point of neutrophil lysis [77]. The observed neutrophil lysis was caspase and ROS-independent, findings incompatible with pyroptosis or NETosis [77]. Rather, the authors suggested that lysis of neutrophils after phagocytosis of *S. aureus* is consistent with programmed necrosis. Indeed, Greenlee-Wacker *et al.* then demonstrated that neutrophils undergo programmed necrosis following phagocytosis of *S. aureus* [98]. Such cytolysis presumably contributes to the pathogenesis of *S. aureus* infections, although direct evidence is still lacking and more work is needed.

## 4. Conclusions

To summarize, *S. aureus* has capacity to produce many molecules directed to inhibit neutrophil functions such as recruitment and phagocytosis. Despite this repertoire of immune evasion molecules, severe invasive *S. aureus* disease is infrequent in otherwise healthy individuals who lack risk factors for infection. In addition, the localized presence of neutrophils is a hallmark of *S. aureus* infections and neutrophils readily phagocytose *S. aureus*. These observations bring into question the role of the *S. aureus* immune evasion molecules in human disease (outside of the at-risk individual). Finally, the possible utility of an opsonophagocytic vaccine for *S. aureus* is confounded by two key features of *S. aureus*-neutrophil interactions: (1) phagocytosis is already efficient in the absence of a vaccine; and (2) neutrophils can undergo rapid lysis after phagocytosis of *S. aureus*. Despite the noted caveats, research in these areas must move forward. Advances in basic research that continue to elucidate the complex interactions between *S. aureus* and the human host will likely offer new inroads into developing more efficacious therapeutic treatments and perhaps, a preventative vaccine.

## Abbreviations

The following abbreviations are used in this manuscript:

PMN: Polymorphonuclear leukocyte
LFA-1: Lymphocyte function-associated antigen-1
PRR: Pattern recognition receptor
PAMP: Pathogen-associated molecular pattern
TLR: Toll-like receptor
ROS: Reactive oxygen species
AMP: Antimicrobial peptide
CHIPS: Chemotaxis inhibiting protein of *S. aureus*
FPR: Formyl peptide receptor
SCIN: Staphylococcal complement inhibitor
SAK: Staphylokinase
SEA: Staphylococcal enterotoxin A
IEC: Immune evasion cluster
Eap: Extracellular adherence protein
Ssls: Staphylococcal-like protein
PSGL-1: P-selectin glycoprotein ligand-1
Efb: Extracellular fibrinogen-binding protein
Ecb: Extracellular complement-binding protein
FLIPr: FPR-like 1 inhibitory protein
Hla: alpha-hemolysin
SpA: *S. aureus* protein A
ScpA: Staphopain A
Sbi: Second binding protein of immunoglobulin
IsdH: Iron-regulated surface determinant protein H
SOD: Superoxide dismutase
MPO: Myeloperoxidase
PSM: Phenol soluble modulin
PVL: Panton-Valentine leukocidin
Hlg: Gamma-hemolysin
